# 40-Year Outcome of Old-School, Non-Surgical Endodontic Treatment: Practice-Based Retrospective Evaluation

**DOI:** 10.3390/dj12040090

**Published:** 2024-04-01

**Authors:** Roland Frankenberger, Stephan Becker, Benedicta Beck-Broichsitter, Susanne Albrecht-Hass, Charlotte J. Behrens, Matthias J. Roggendorf, Andreas Koch

**Affiliations:** 1 Department of Operative Dentistry, Endodontics, and Pediatric Dentistry, Medical Center for Dentistry, University Medical Center Giessen and Marburg, 35392 Marburg, Germany; dr.roggendorf@mac.com (M.J.R.); kochan@staff.uni-marburg.de (A.K.); 2 Prof. Becker & Kollegen, Kehdenstrasse 2-10, 47803 Kiel, Germany; praxis@becker-mkg.de (S.B.); behrens@becker-mkg.de (C.J.B.); 3 Clinic for Oral and Maxillofacial Surgery, Stuttgart City Clinic, Kriegsbergerstr. 60, 70174 Stuttgart, Germany; b.beck-broichsitter@klinikum-stuttgart.de; 4 Private Practice, Blumenweg 2, 24226 Heikendorf, Germany; info@zahnarztpraxis-heikendorf.de

**Keywords:** endodontic treatment, root canal disinfection, root canal preparation, root canal obturation, clinical outcome, root canal post, post-endodontic restoration

## Abstract

(1) Background: Non-surgical endodontic treatment has been shown to be clinically successful; however, clinical long-term data are scarce. This practice-based retrospective clinical investigation evaluated endodontic outcomes over 40 years and identified relevant clinical co-factors. (2) Methods: Two experienced dental practitioners in two different private dental practices treated 174 patients with 245 teeth from 1969 to 1993. After root canal obturation, either a new direct restoration (amalgam, resin composite, or glass-ionomer cement) or the re-cementation of a pre-existing prosthetic restoration or renewal of prosthetic restoration followed. Metal posts (operator A) or metal screws (operator B) were inserted when coronal substance loss was significant. The primary outcome (i.e., tooth survival) was achieved when the endodontically treated tooth was, in situ, painless and had full function at the end of the observation period. A secondary outcome, the impact of different prognostic factors on survival rate, was evaluated. (3) Results: The overall mean survival was 56.1% of all treated teeth after 40 years of clinical service, resulting in an annual failure rate of 1.1%. Most investigated clinical co-factors (jaw, tooth position, intracanal dressings, post/screw placement, and gender) showed no significant influence on survival. (4) Conclusions: Even with materials and techniques from the 1970s and 1980s, successful root canal treatment was achievable. Except for post-endodontic restorations, most of the evaluated factors had no significant influence on the clinical long-term survival of root canal-treated teeth.

## 1. Introduction

Although both caries decline and improved caries excavation regimens potentially protect pulp vitality [1,2,3], successful endodontic treatment is still a key factor in conservative dentistry when irreversible pulpitis is present [4,5]. Unfortunately, today, a significant shift toward systematic reviews is seen in the dental literature, which is, on the one hand, desirable, but on the other hand, it is questionable when it comes to the phenomenon that collected data are more frequently cited than original research [6,7]. This inflation of “literature papers” may tempt young researchers to primarily use their computers instead of heading to a lab or chair where real data are produced [8]. Moreover, in many countries, prospective clinical trials are increasingly impeded by sprawling bureaucracy as well as inappropriate ethical concerns and burdens [9,10]. All of these factors make clinical research more and more expensive, consequently less attractive for the dental industry, and finally, less accomplishable in general [10].

Successful pulp treatment is strongly correlated with patients’ quality of life because tooth preservation is a major factor for human well-being [11,12,13]. However, limited clinical data represent a weak scientific basis for clinical decision-making [14]. Therefore, more clinical research in endodontics has been repeatedly requested [15]. Along with randomized trials as the gold standard, prospective cohort studies [8,15] have also been described as promising [15]. Furthermore, the inclusion of several variables combined with multivariate statistics are fundamental prerequisites to reduce the risk of confounding bias [16]. On the other hand, large-scale prospective cohort studies involving multiple variables are extremely time-consuming and expensive [8,15], and it is well-known that once reliable data from these studies are available, the assessed biomaterials under investigation may not be on the market anymore [17]. These are the main reasons why very few endodontic prospective long-term trials have been published [17,18,19]; additionally, they suffer from relatively short follow-up periods. Real long-term data are, therefore, desired for both patient information and public health as well as insurance issues [4,15]. Although retrospective studies still have an inferior impact compared with randomized clinical trials, they still are of some value to the clinician.

Besides endodontic treatment alone, the quality of post-endodontic restoration is an important factor for the clinical long-term survival of endodontically treated teeth as well [20]. It is not clear whether the quality of endodontic treatment or post-endodontic restoration is more important here [20] because both factors seem to equally contribute to clinical success [21]. Facing treatment outcomes from older decades, like in the present investigation, it is interesting that almost no cofactors that are relevant today were investigated before, i.e., today’s evidence about post-endodontic restorations [3,21,22,23,24,25] was not available in the 1980s when metal posts and screws were the clinical standard, as were amalgam restorations. By contrast, when direct resin composite restorations were used in former times, dentin bonding was not available at all, so all dentin areas were covered with a thick conventional lining of phosphate cement. This means that in every single adhesive approach documented, only the enamel margins were really bonded.

The present study retrospectively evaluated the 40-year outcomes of endodontic treatment of 174 patients and 243 teeth having been endodontically treated by two different dentists in the 1970s and 80s.

## 2. Materials and Methods

Endodontic treatments in the present retrospective study were performed by two experienced dental practitioners (P1: Albrecht, P2: Behrens) at two different private dental practices in Kiel, Germany. P1 treated 73 patients/107 teeth, and P2 treated 101 patients/138 teeth. The study protocol and informed consent procedures were approved by a local ethics committee (University of Kiel; Ref. No. 474/18); prior to the investigation, all patients provided informed consent. All endodontic treatments were performed from 1969 to 1993. Evaluations were carried out by the two practitioners until 2018 involving long-term periods of 25–49 years, with an average observation period of 40 years.

The paper was written in consideration of STROBE guidelines. Patients were included if a primary or secondary endodontic treatment was indicated (irreversible pulpitis, non-vital teeth, or no re-treatment) and only when endodontological standards, such as rubber dam and field isolation, were possible. Furthermore, teeth were only included when post-operative restoration was realistically possible. Teeth with any periodontal loosening, internal or external root resorptions, or root fractures of any kind were excluded. Endodontic treatment procedures were carried out according to contemporary methods of the respective period. In vital extirpation cases, root canals were prepared with manual Hedstrom files, rinsed with H_2_O_2_, dried with paper points, and filled with gutta-percha and contemporary sealers using a lateral compaction technique with metal spreaders and moderate pressure. Prior to preparing root canals with endodontic instruments, patients were clinically and radiographically examined. Clinical examination included an evaluation of tooth position, pre-existing restoration, periodontal status, pain symptoms, vitality status, and sensitivity to percussion. The presence and depth of caries as well as the presence and size of apical radiolucency were evaluated radiographically. A rubber dam was always applied when possible. The working length was determined radiographically to be set 1 mm short of the radiological apex. Finally, a radiograph was taken to check the quality/homogeneity and distance of the root canal filling to the individual apex. In non-vital teeth, 3% sodium hypochlorite was used as a disinfectant, and when the treatment required several sessions, intracanal medication (ChKM/Speiko, Calxyl/OCO) was applied.

Subsequent to root canal obturation, all teeth were either restored with a new direct restoration (amalgam, resin composite, or glass ionomer cement), recementation of pre-existing prosthetic restorations, or manufacturing of new indirect prosthetic restorations. Cast metal posts and intracanal screws were inserted when significant loss (>50%) of coronal tooth structure required additional intracanal retention. In cases of failed or lost post-operative restorations, endodontically treated teeth were newly restored at different times in the following years.

In the period following the completion of endodontic and restorative treatments, all patients joined an individual monitoring program for clinical reevaluation, including subsequent control radiographs. Depending on the associated findings, endodontically treated teeth received periodontal therapy, endodontic re-treatment, or apicectomy in due course. Both operators investigated 245 teeth that were endodontically treated in the period of 1969–1993. Pre-, intra-, and post-operative data and possible prognostic factors with corresponding data were collected in Excel sheets (Table 1 and Table 2). The primary outcome, tooth survival, was achieved when the endodontically treated tooth was in situ, painless, had full function, and was not subject to apicectomy [19]. The secondary outcome was to investigate the impact of potential prognostic factors on survival rates. Due to its retrospective character, the study did not suffer dropouts because only patients who were available until the final examination were included in the protocol.

The statistical analysis of tooth survival was performed by a Kaplan–Meier survival test considering the date of tooth extraction. Reasons for extraction were advanced periodontal disease, root/crown fracture, untreatable caries, and periapical flare-up. Tooth-related variables to evaluate possible prognostic factors were compared by log-rank, Mantel–Haenszel, and Wilcoxon tests. Prism/GraphPad (Insight Partners, GraphPad Holdings, LLC., Los Angeles, CA, USA) was used for statistical analysis, and the level of significance was set at *p* ≤ 0.05.

## 3. Results

### 3.1. Clinical Outcomes for Practitioner A

#### 3.1.1. Overall Tooth Survival

**Figure 1:**
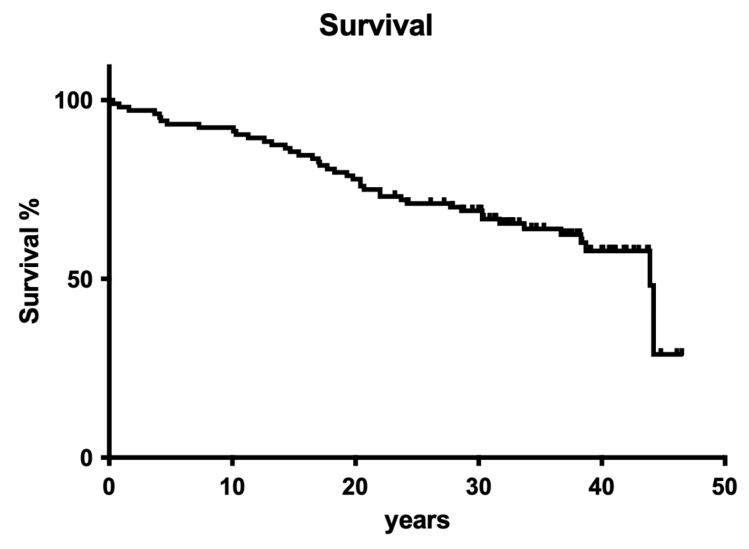
Overall clinical survival of teeth treated by operator A.

Overall tooth loss after 40 years was 43.9%. The observed extraction rate was homogeneously distributed over the complete observation period, so no cumulative incidents, as seen in many other clinical trials, occurred (Figure 1).

#### 3.1.2. Influence of Jaw

**Figure 2:**
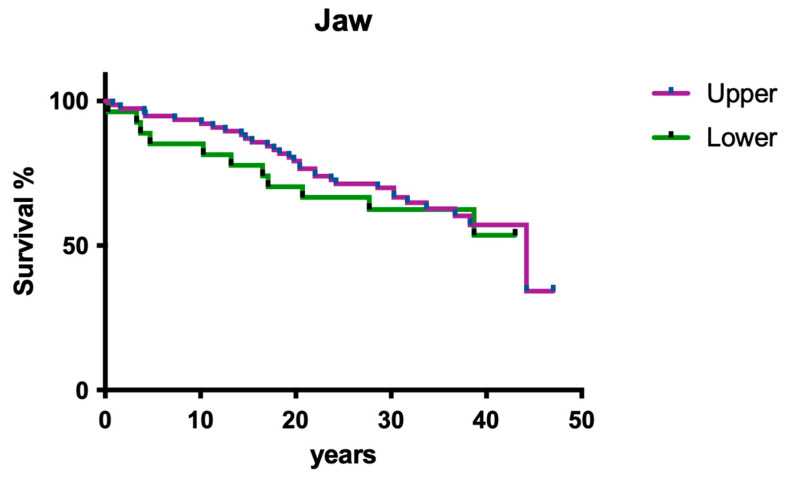
Overall clinical survival in upper and lower jaws.

Jaw position had no significant impact on overall survival (Figure 2; *p* > 0.05).

#### 3.1.3. Influence of Tooth Position

**Figure 3:**
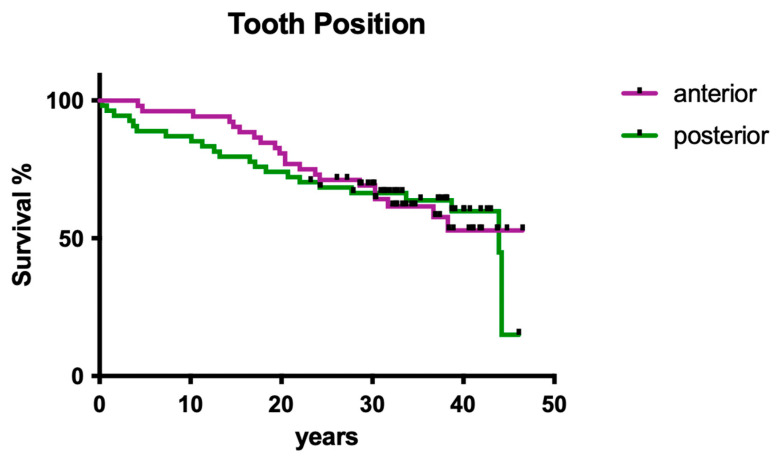
Overall clinical survival of anterior vs. posterior teeth treated by operator A.

Tooth position (i.e., anterior vs. posterior) had no significant influence on overall survival (Figure 3; *p* > 0.05).

#### 3.1.4. Influence of Root Canal Infection

**Figure 4:**
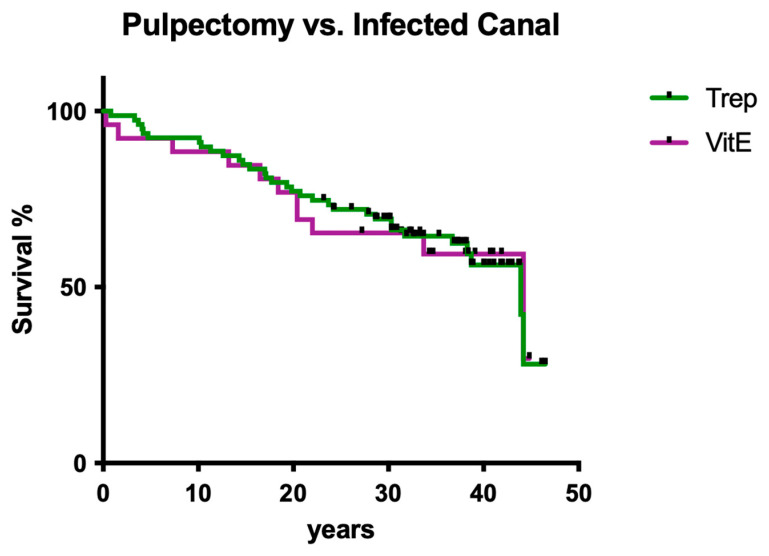
Overall clinical survival of teeth with different infections of the root canal system.

Infection status (pulpectomy vs. infected canal) had no significant influence on overall survival (Figure 4; *p* > 0.05).

#### 3.1.5. Influence of Post Insertion

**Figure 5:**
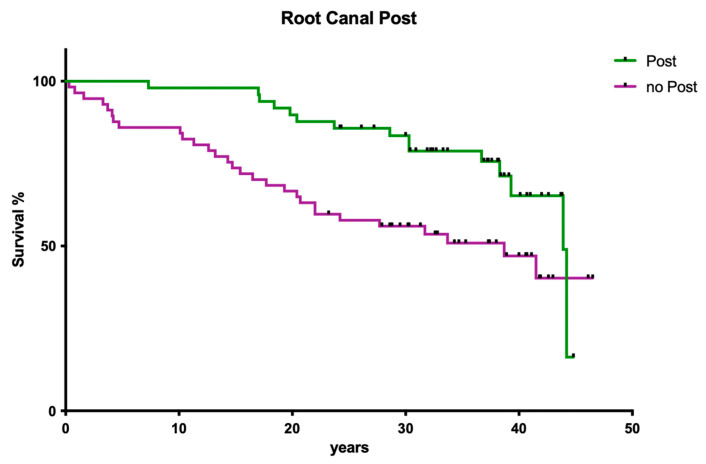
Clinical survival of teeth with posts vs. those with no posts after endodontic restoration.

The presence of post-endodontically inserted root canal posts had no influence on clinical long-term success (Figure 5; *p* > 0.05).

#### 3.1.6. Influence of Periodontal Ligament Space

**Figure 6:**
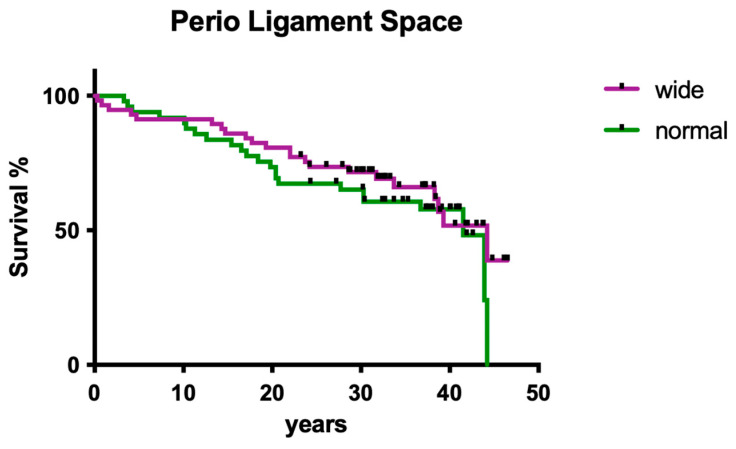
Clinical survival of teeth with differently wide perio ligament spaces.

The radiographically evaluated width of the periodontal ligament space did not show a significant influence on clinical outcome (Figure 6; *p* > 0.05).

#### 3.1.7. Influence of Gender

**Figure 7:**
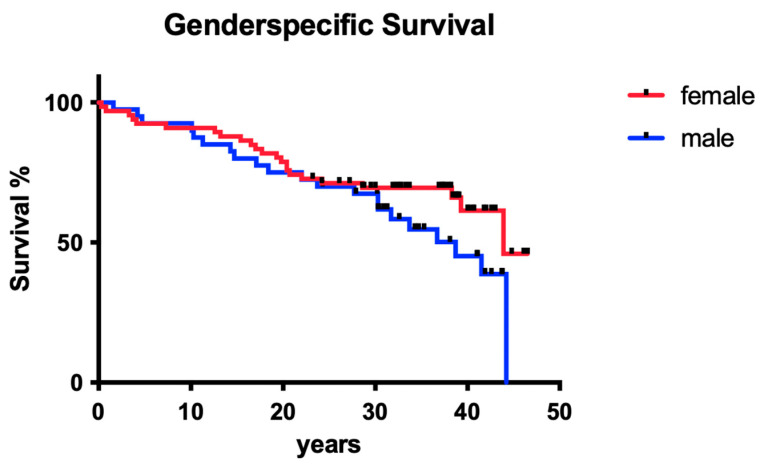
Clinical survival of teeth in males vs. females.

The sex of the patients did not show a significant influence on long-term tooth survival (Figure 7; *p* > 0.05).

#### 3.1.8. Influence of Working Length

**Figure 8:**
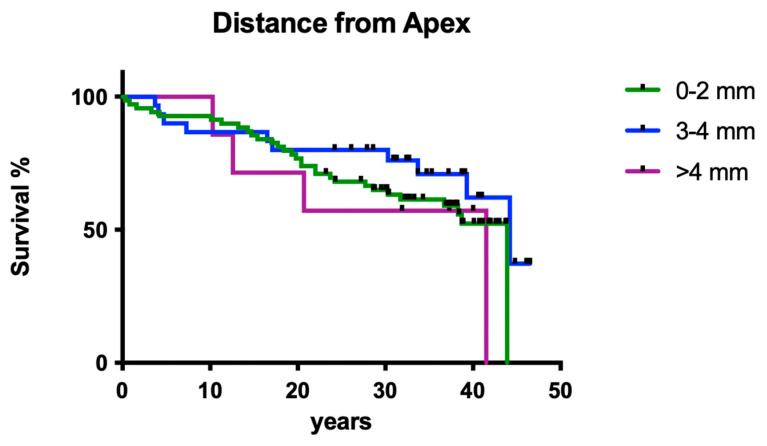
Clinical survival of teeth with different working lengths. It is classified as normal (0–2 mm), hypoinstrumented (3–4 mm), and too short (≥4 mm).

The measured distance of the root canal filling to the radiological apex did not have a significant influence on the clinical long-term survival of endodontically treated teeth (Figure 8; *p* > 0.05).

#### 3.1.9. Influence of Intracanal Dressing Changes

**Figure 9:**
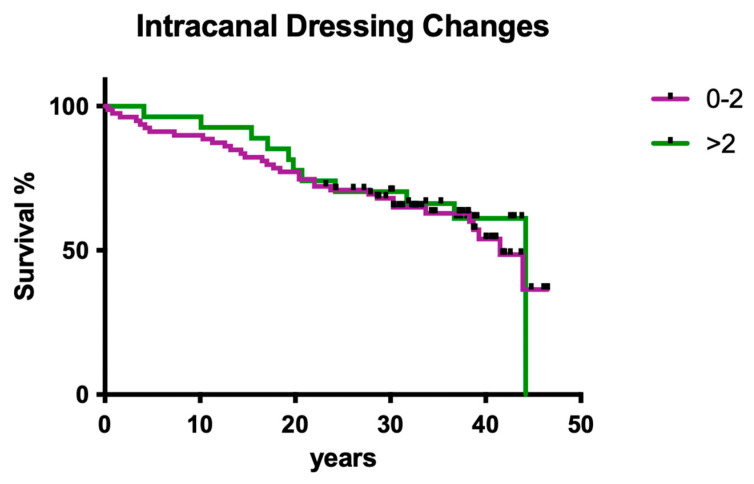
Clinical survival of teeth with different frequencies of root canal dressing changes.

The number of intracanal medicament dressing changes did not have a significant effect on clinical outcome (Figure 9; *p* > 0.05).

### 3.2. Clinical Outcome Practitioner B

#### 3.2.1. Overall Survival

**Figure 10:**
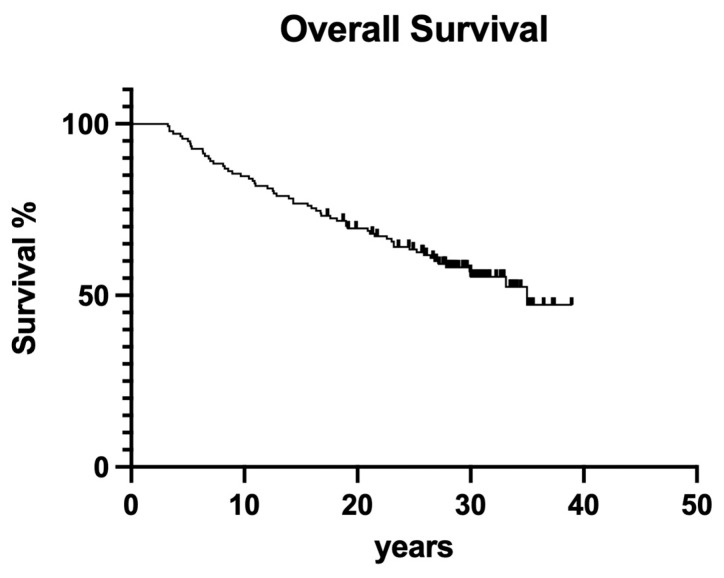
Overall clinical survival of operator B.

The overall clinical survival of operator B was slightly higher than that of operator A, but the difference was not statistically significant (Figure 10; *p* > 0.05).

#### 3.2.2. Jaw

**Figure 11:**
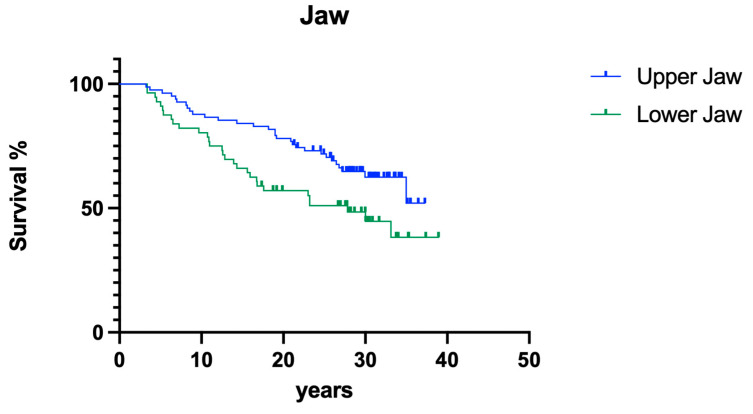
Overall clinical survival of teeth in different jaws.

Jaw position had no significant impact on overall survival (Figure 11; *p* > 0.05).

#### 3.2.3. Number of Intracanal Dressing Changes

**Figure 12:**
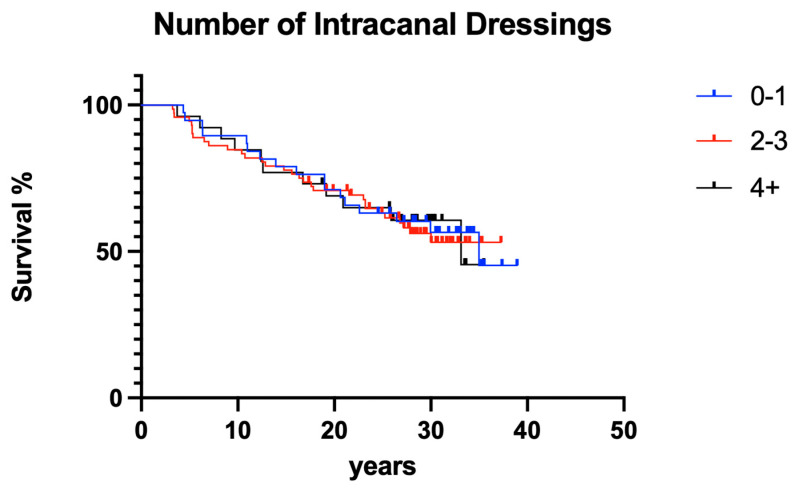
Overall clinical survival of teeth with different numbers of intracanal dressings.

The number of intracanal medicament dressing changes did not have a significant effect on clinical outcome (Figure 12; *p* > 0.05).

#### 3.2.4. Screws

**Figure 13:**
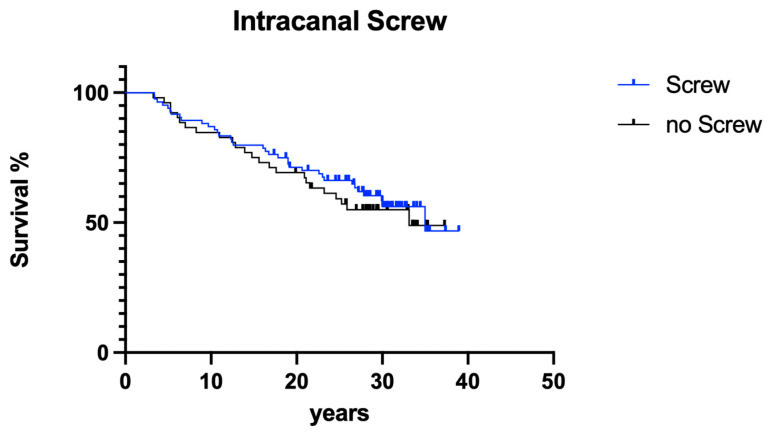
Overall clinical survival of teeth with screws vs. those with no screws.

The presence of post-endodontically inserted root canal retention screws had no influence on clinical long-term success (Figure 13; *p* > 0.05).

#### 3.2.5. Post-endodontic Restoration

**Figure 14:**
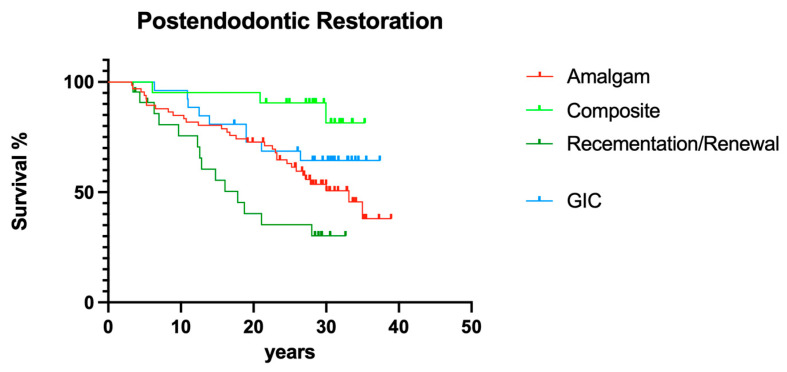
Clinical survival of teeth with different post-endodontic restorations.

Different post-endodontic restorations showed a significant effect on overall survival; direct resin composite restorations led to fewer failures than prosthetic restorations (Figure 14; *p* < 0.05).

### 3.3. Interoperator Comparison

**Figure 15:**
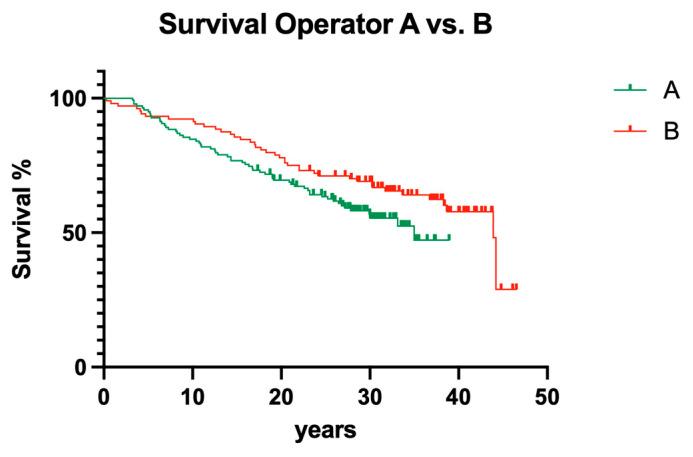
Clinical survival of teeth with different operators.

Between the operators involved in the present study, no significant influence could be computed (Figure 15; *p* > 0.05).

### 3.4. Overall Survival

**Figure 16:**
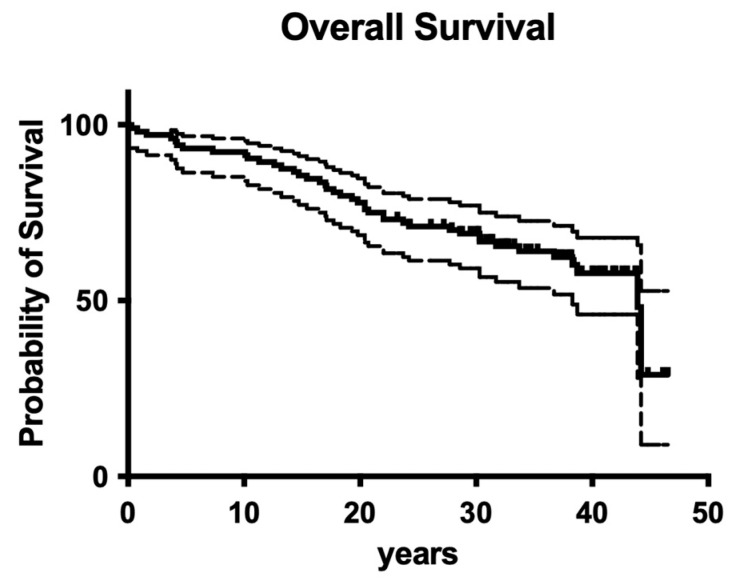
Clinical survival of both operators.

Overall survival for the whole retrospective study was 56.1% after 40 years of clinical service (Figure 16).

## 4. Discussion

Although there are only a few clinical long-term studies available, it is well-known from the literature in the field that endodontic treatments are successful and thus responsible for long-term tooth preservation [4,18,19]. After the 1990s, enormous progress in root canal preparation techniques was documented, especially after more flexible NiTi instruments were developed [17,26,27]. However, up to now, it is still not fully understood whether the old-fashioned root canal treatment regimens of the past were less successful than recent advanced techniques. When using quite stiff steel instruments, it may be a primary concern that routine deviation of the root canal system is produced and in turn may lead to significantly less overall success of endodontic treatments. As a matter of fact, it has to be taken into account that in the presented cases, several deviations of root canal systems may have occurred. Besides root canal preparation issues, obsolete intracanal dressings and sealer materials were used back then. To make it clear, today, no dentist would even think of using materials like ChKM simply because much better alternatives in any indication exist [5,28,29]. However, material-specific toxicity, primarily with ChKM, may have played a significant role in counterbalancing less effective root canal shaping by simply acting more as a bactericidal. Another point may be that adhesive fatigue phenomena, which have also been described previously in post-endodontic restorations, were not present. Therefore, the aim of the present retrospective evaluation was to investigate 1970/80s-style endodontic treatment, relating its outcomes over more than four decades.

**Figure 17:**
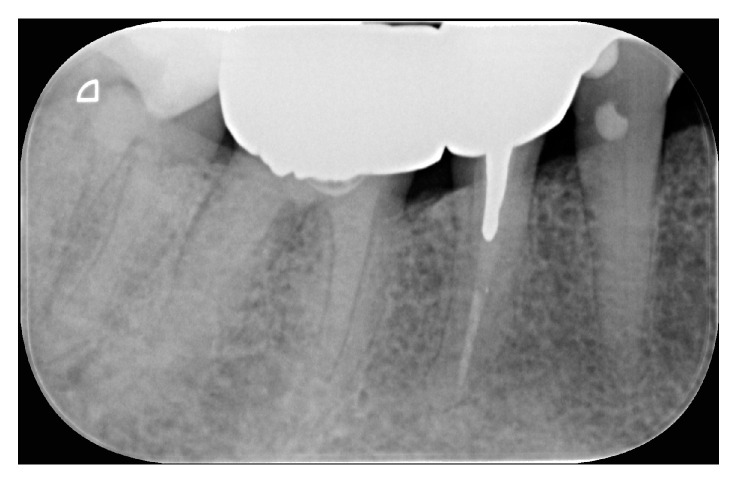

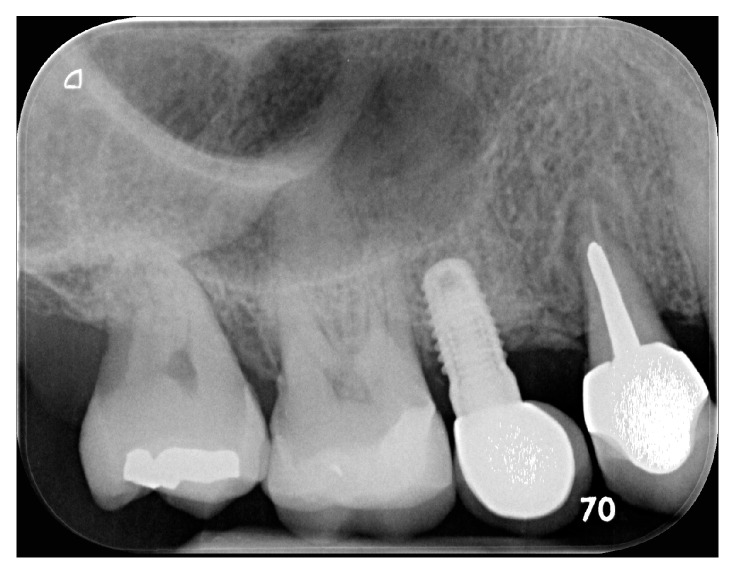
Radiographic outcome.

To cut a long story short, old-fashioned, non-surgical endodontic treatment was also successful over the observation period of 40 years. From today’s standpoint, with significantly improved root canal instruments providing better cleaning and shaping and less deviation [27], it is astonishing that more than 50% of endodontically treated teeth were still in function until the end of the observation period (Figure 16 and Figure 17). Although materials and techniques that are regarded as obsolete today were used, the final documented outcome was excitingly good. So, when materials and techniques are obviously less decisive factors for clinical outcome, what is the key to clinical success? A closer look into the treatment protocols of the available documentation exhibits two important aspects: (1) both operators exhibited a high level of experience, a factor that has also been shown to be of predominant importance in other fields of dentistry [30], and (2) only teeth with zero periodontal loosening have been included in the study because both operators also had a strong background in periodontology. So, according to the data of the present evaluation, both factors seem to positively influence the long-term outcome of endodontic treatments. The same may be true for rubber dam application, which is liked by many patients when swallowing fewer root canal irrigants and represents a high treatment standard in endodontics.

Regarding the estimated co-factors for clinical success, it was shown that the gender of the patients, the jaw, tooth position (anterior vs. posterior), and the number of intracanal dressing/medication changes did not have a significant impact on clinical outcome. Compared with the literature in the field of endodontology, the most surprising finding of the present study was clearly that the distance from the root filling to the radiographical apex apparently did not affect the clinical outcome. This may be justified by the fact that many pulpectomies have been performed as primary endodontic treatment with less need for disinfection protocols [4,5,17]; however, it remains surprising. Of course, a general limitation of the present study may be the patient number, which may also be a factor explaining why for these particular questions, no statistically significant difference was computed.

It has been repeatedly reported that the quality of the individual post-endodontic restoration is an important factor for long-term tooth survival [21,23,24,25]; sometimes it was even regarded as more important than the quality of the root canal obturation itself [20]. Also in the present trial, significant differences between the involved post-endodontic restorations were computed. Although adhesive technologies were neither fully understood nor extremely advanced in the 1980s, the outcome of resin composite fillings as post-endodontic restorations was surprisingly good. However, normally, smaller defects receive more direct resin composite restorations [24], so this observation may just be correlated to primary defect size and, therefore, be somewhat questionable retrospectively. Due to underdeveloped adhesive regimens in the 1980s, it is feasible that larger defects were not restored with resin composites, which means that the outcome of these particular restorations may be overestimated compared with conventional prosthodontic restorations. Although screw-retained post-endodontic buildups have been reported to fall short [31], the present data revealed no difference between screws and posts; however, every single post was luted with conventional cements and not fully adhesively, as is standard today [23,24,25]. This may explain the similar results when it comes to screws vs. posts for post-endodontic buildup [23,24,25].

Nevertheless, facing the array of clinical variables that were simply not known in the 1980s, combined with the fact that the flexibility of root canal instruments was underdeveloped as well [26], the overall outcome of the present retrospective investigation is surprisingly good. This also has a significant impact on the quality of life of the investigated group of patients [12]. A final critical question remains: although we know so much more today in almost every aspect investigated here [23], and although we definitely have access to much more advanced materials, methods, and protocols [21], are we really more successful today in clinical endodontics [16]? Maybe the old wisdom in endodontology is still true: it is less important what you apply to the root canal—it is more important what you remove from the root canal.

## 5. Conclusions

Endodontic treatment of root canals filled in the 1980s showed a good clinical outcome over 40 years of clinical service. Both the experience of the operators and narrow/strict indications seem to be decisive factors for clinical success.

## Figures and Tables

**Table 1:** Variables and co-variables of operator 1.

Variables (P1)		Endodontically Treated Teeth	Extractions
Preoperative			
		n = 107	n = 46
Gender	Male (29)	66	
	Female (44)		
Tooth location	Upper jaw	77	
	Lower Jaw	27	
	Anterior	52	
	Posterior	54	
Coronal caries		63	31
Root caries		4	2
Sensitivity to percussion	Yes	75	30
	No	32	16
Vitality status	Vital	29	16
	Non-vital	75	30
Presence of apical radiolucency	Yes	45	27
	No	62	19
Periapical space	Normal	49	23
	Enlarged	58	23
Tooth function	Distal tooth in quadrant	13	4
	Bridge in abutment	10	9
	Double proximal contact	84	33
Distance of root canal filling to apex	0–2 mm	59	27
	2–3 mm	31	12
	≥4 mm	16	7
Number of intracanal medications	0	35	14
	1	21	11
	2	24	9
	3	19	10
	4 or more	8	2
Post	Yes	49	17
	No	58	29

**Table 2:** Variables and co-variables of operator B and comparisons of A vs. B.

Variables (P2)		Endodontically Treated Teeth	Extraction
Pre-operative			
		n = 138	n = 61
Gender	Male		
	Female		
Deep caries		11	6
Vitality status	Vital	46	23
	Non-vital	60	23
	No data	32	15
Pain symptoms	Yes	11	5
	No data	127	56
Sensitivity to percussion	Yes	137	61
	No	1	0
Operator	P1	104	42
	P2	138	60
	P1 anterior	52	21
	P1 posterior	54	22
	P2 anterior	30	9
	P2 posterior	108	51
Tooth location	Upper jaw	82	30
	Lower Jaw	56	30
	Anterior upper jaw	30	9
	Posterior upper jaw	52	21
	Posterior lower jaw	56	30
Number of intracanal medications	0–1	38	17
	2–3	72	31
	≥4	26	11
Duration from trepanation to root canal filling	0	18	10
	1–14 days	10	4
	15–28 days	33	12
	>28 days	75	34
Post	Yes	86	37
	No	52	24
Restoration	Amalgam	66	33
	Composite	21	3
	Recementation/renewal of prosthetic restoration	22	14
	Glass ionomer cement	26	9
Periodontal therapy	Yes	30	13
	No	108	47

## Data Availability

Data are available upon reasonable request.
